# Supplementary material for: Inhibition of Cxcr4 Disrupts Mouse Embryonic Palatal Mesenchymal Cell Migration and Induces Cleft Palate Occurrence
Source: Int J Mol Sci. 2023 Aug 13;24(16):12740. doi: 10.3390/ijms241612740 (PMC10454820; doi:10.3390/ijms241612740)
Supplement: Supplementary file 1 [file ijms-24-12740-s001.zip › ijms-2529886-supplementary.pdf]

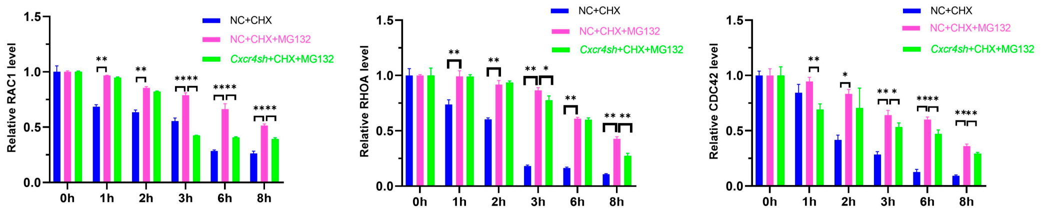

Supplementary Figure S1. Quantitative analyses of RAC1, CDC42, RHOA degradation

Quantitative analyses of RAC1, CDC42, RHOA degradation in the NC group and *Cxcr4sh* group in the presence of CHX and MG132 are shown

(n = 3). *P < 0.05, **P < 0.01.
